# Automated yeast cultivation control using a biosensor and flow cytometry

**DOI:** 10.1093/jimb/kuae039

**Published:** 2024-10-18

**Authors:** Raquel Perruca Foncillas, Sara Magnusson, Basel Al-Rudainy, Ola Wallberg, Marie F Gorwa-Grauslund, Magnus Carlquist

**Affiliations:** Division of Applied Microbiology, Department of Chemistry, Lund University, SE-22100 Lund, Sweden; Division of Applied Microbiology, Department of Chemistry, Lund University, SE-22100 Lund, Sweden; Division of Chemical Engineering, Department of Process and Life Science Engineering, Lund University, SE-22100 Lund, Sweden; Division of Chemical Engineering, Department of Process and Life Science Engineering, Lund University, SE-22100 Lund, Sweden; Division of Applied Microbiology, Department of Chemistry, Lund University, SE-22100 Lund, Sweden; Division of Applied Microbiology, Department of Chemistry, Lund University, SE-22100 Lund, Sweden

**Keywords:** automated real-time flow cytometry, fermentation, control strategy, heterogeneity, synthetic biology

## Abstract

Effective microbial bioprocessing relies on maintaining ideal cultivation conditions, highlighting the necessity for tools that monitor and regulate cellular performance and robustness. This study evaluates a fed-batch cultivation control system based on at-line flow cytometry monitoring of intact yeast cells having a fluorescent transcription factor-based redox biosensor. Specifically, the biosensor assesses the response of an industrial xylose-fermenting *Saccharomyces cerevisiae* strain carrying the *TRX2*p-*yEGFP* biosensor for NADPH/NADP+ ratio imbalance when exposed to furfural. The developed control system successfully detected biosensor output and automatically adjusted furfural feed rate, ensuring physiological fitness at high furfural levels. Moreover, the single-cell measurements enabled the monitoring of subpopulation dynamics, enhancing control precision over traditional methods. The presented automated control system highlights the potential of combining biosensors and flow cytometry for robust microbial cultivations by leveraging intracellular properties as control inputs.

**One-Sentence Summary:**

An automated control system using flow cytometry and biosensors enhances microbial bioprocessing by regulating cellular performance in response to the environmental stressor furfural.

## Graphical Abstract

**Graphical Abstract:**
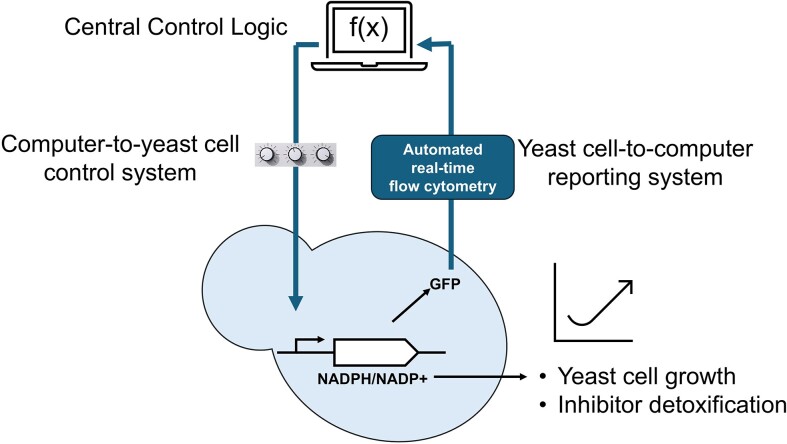

## Introduction

Optimal cultivation conditions are a prerequisite for the success of robust and efficient bioprocesses. Therefore, the use of monitoring strategies capable of real-time measurements and adjustments of environmental cues is crucial. Bioreactors are often equipped with monitoring systems that allow them to maintain optimal environmental conditions such as pH, temperature, or dissolved oxygen (Bertaux et al., 2022). Unfortunately, this is generally not the case for other relevant parameters such as cell biomass, metabolic state, or product formation, for which measurements often rely on manual sampling and analysis, limiting their applicability as monitoring parameters (Gargalo et al., 2022).

Advances have been made in recent years in developing real-time sensors based on spectroscopic techniques such as ultraviolet-visible spectroscopy, near- and mid-infrared spectroscopy, and Raman spectroscopy (Gargalo et al., 2022). However, these methods are based on the measurement of extracellular metabolites, while little is known about intracellular processes that can affect the efficiency of the cultivation. To shed some light on it, non-invasive fluorescent biosensors can be utilized. For example, the *TRX2*p-*yEGFP* biosensor (Zhang et al., 2016) has been utilized to study the response of *Saccharomyces cerevisiae* to the presence of lignocellulosic inhibitors such as furfural (Zhang et al., 2016; Torello Pianale et al., 2022; Perruca Foncillas et al., 2023). TRX2 encodes a cytoplasmic thioredoxin isoenzyme, which is a crucial component of the thioredoxin system and regulated by the transcription factor Yap1p in response to oxidative stress. The *TRX2p-yEGFP* biosensor utilizes a reengineered version of the native Trx2p promoter with increased number of Yap binding sites and the upstream activating sequence of the TRX2p promoter, significantly enhancing its dynamic range. The biosensor employs yEGFP (yeast-enhanced green fluorescent protein) to detect NADPH/NADP+ (nicotinamide adenine dinucleotide phosphate hydrogen/nicotinamide adenine dinucleotide phosphate) perturbations induced by oxidative agents like furfural. Furfural is known to cause NADPH deficiency due to reduction to furan methanol by NADPH-dependent reductases and reactive oxygen species (ROS) formation (Allen et al., 2010). The presence of furaldehyde is a major challenge for the commercialisation of lignocellulosic bioethanol. With the use of the *TRX2*p-*yEGFP* biosensor, the redox state of the cells can be indirectly monitored by measuring the fluorescence intensity of the cells.

In parallel to the development of *in vivo* biosensors, flow cytometry (FCM) has gained interest as an analysis tool as it can be used for rapid quantification of cellular fluorescence at the single-cell level (Shapiro, 2003). FCM analysis can be performed with high sampling frequency with automated samplers such as onCyt (Besmer et al., 2014), which in combination with an automated data processing pipeline can provide detailed information on population heterogeneity dynamics (Rao et al., 2023). However, the transition from the monitoring capability of an automated FCM to a reactive FCM that utilizes the results obtained from the FCM analysis to generate a response accordingly in a control system remains challenging (Delvigne & Martinez, 2023; Heins et al., 2022).

In this study, we demonstrated the application of an automated FCM system for real-time monitoring of the *TRX2*p-*yEGFP* response in *S. cerevisiae* to furfural in a fed-batch cultivation process. To control the substrate feed pump, we built a control module capable of dynamically regulating the addition of substrate as a function of the biosensor response. The developed system was initially assessed in fed-batch cultivations and further applied to the propagation of yeast in a bioethanol production process.

## Materials and Methods

### Strains and Media

The industrial *S. cerevisiae* strain TMBRP011 carrying the redox imbalance biosensor *TRX2*p-*yEGFP* was used (Perruca Foncillas et al., 2023). Its cultivation was performed in yeast peptone (YP) medium containing 20 g/L peptone and 10 g/L yeast extract supplemented with the appropriate carbon source.

### Fixed Feed Rate Fed-Batch Cultivations

The strain TMBRP011 was pre-cultivated in two steps. Initially, TMBRP011 was grown overnight in a 50 mL Falcon tube with 5 mL YPD (YP + 20 g/L glucose). Then, this first pre-culture was used to inoculate a second 50 mL Falcon tube containing 5 mL YPD to an initial optical density (OD) of 0.6 and grown for another 4.5 hr. The cultivations were performed at 30°C and 180 rpm.

The following cultivations were performed in a 500 mL baffled shake flask incubated at 30°C in a shaking water bath. The shake flask contained 50 mL YP medium supplemented with 40 g/L glucose and 40 g/L xylose and the initial OD was 0.1.

After glucose depletion was confirmed by high-performance liquid chromatography (HPLC) analysis, the feeding phase was initiated. A peristaltic pump (Alitea U1/4–4R) was utilized to introduce 150 mL of feed at a fixed feed rate (F-FR) of 6.24 mL/hr. The feed consisted of YP medium supplemented with 20 g/L glucose, 20 g/L xylose, and 6.9 g/L furfural. Samples were collected for OD and HPLC analysis. Fluorescence intensity was monitored throughout the cultivation with an automated FCM system.

### Sensor-controlled Feed Rate Fed-Batch Cultivations

The experimental setup utilized in the F-FR fed-batch cultivations was further developed to implement a control strategy. In this case, a computer program was developed to control the feed rate of an additional furfural solution into the shake flask based on the results of the FCM analysis. The additional solution contained 50 g/L furfural.

The computer program was written in Python 3.7.9 and ran on a Windows 7 computer. The “FlowCytometryTools” package (Yurtsev & Friedman, 2015) was used to extract data from the flow cytometry standard (FCS) files. The Python code is available in the following GitHub repository: MicrobialEngineeringGroupTMB (2024), Scripts-for-Automated-yeast-propagation-control-using-a-biosensor-and-flow-cytometry (Version #1). https://github.com/MicrobialEngineeringGroupTMB/.

### Anaerobic Fermentations on Hydrolysate

After the cells were propagated in F-FR or sensor-controlled feed rate (SC-FR) fed-batch cultivations, the equivalent of 3 g cell dry weight/L of cells was used to inoculate serum vials containing 50 mL of YP medium supplemented with 71% (v/v) of wheat straw hydrolysate, whose composition has been described elsewhere (Perruca Foncillas et al., 2023). Sugars were complemented to reach 20 g/L of glucose and 20 g/L of xylose. The vials were flushed with N_2_ for at least 40 min and sealed with rubber stoppers. The incubation was performed in a shaking incubator at 30°C and 180 rpm.

### Automated FCM

Flow cytometry analysis was performed using onCyt (onCyt, Switzerland) coupled to an Accuri C6+ (BD Biosciences, NJ, USA) as previously described (Perruca Foncillas et al., 2023). Samples were automatically taken every 30 min from the shake flask, diluted 100 times in phosphate buffer saline at pH 7.4, stained with 10 µg/mL propidium iodide (PI), and incubated for 5 min. A fixed volume of 35 µL of sample was run at a flow rate of 35 µL/min. Sterile-filtered air (0.22 µm) was used to push the culture left in the sampling line back into the shake flask to avoid cell growth in the sampling line that could clog it, as well as to recover culture volume. Water was run for 4 min in between the samples as a cleaning step. Flow cytometry data were analysed using FlowJo as described previously (Perruca Foncillas et al., 2023), or with the method developed in this study using the “FlowCytometryTools” package (Yurtsev & Friedman, 2015).

### Analytical Methods

An Ultrospec 2100 pro UV/Visible spectrophotometer (Amersham Biosciences, Buckinghamshire, UK) was used to measure the OD and estimate the yeast biomass concentration of the samples. A linear correlation between OD and cell dry weight (CDW) was used to transform OD values into CDW (g/L). Extracellular metabolites such as glucose, xylose, glycerol, acetate, lactate, ethanol, HMF, and furfural were analysed using a Waters HPLC system (Milford, CT, USA) equipped with Phenomenex Rezex ROA-Organic Acid column operating at 60°C. Isocratic 5 mM sulfuric acid was used as mobile phase and the flow rate was maintained at 0.6 mL/min.

## Results

### Development of an Automated Control Strategy Using FCM

**Fig. 1.**
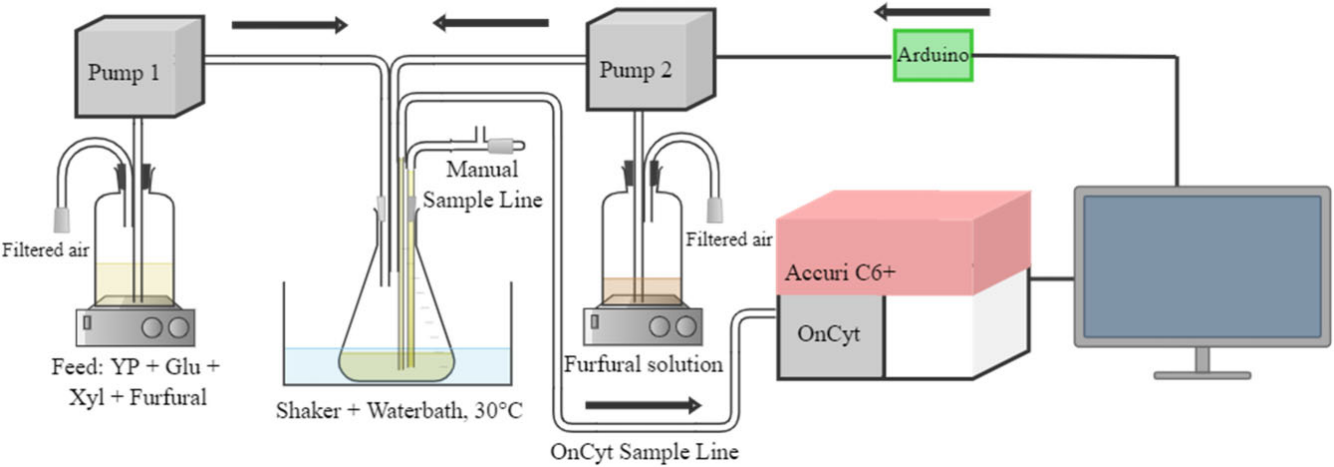
Schematic diagram of control strategy. The cell propagation is performed in fed-batch mode in a shake flask placed on a shaking water bath at 30°C. This flask is connected to the primary feed containing rich medium supplemented with furfural which is pumped at a constant feeding rate throughout the cultivation. Samples are taken by the automatic sampler (onCyt) and analysed by the flow cytometer (Accuri C6+). The biosensor response is analysed by the computer program which controls the feeding rate of the secondary pump accordingly using an Arduino for communication. The secondary feed contains a concentrated furfural solution.

The *TRX2*p-*yEGFP* biosensor, measured through at-line FCM, allows the assessment of the yeast response to the lignocellulosic inhibitor furfural (Perruca Foncillas et al., 2023). Analysis of yeast populations during cultivation may be problematic due to differences in the potential skew of distributions obtained with FCM. Another problem is the higher cell densities in combination with too long down time between samples may cause clogging of the tubing. To address these issues, the onCyt was programmed to do different number of dilution steps in the beginning of the cultivation than in the end, thus having more similar number of events recorded per sample. At higher cell densities in the later stages of cultivation, clogging of tubes may potentially be another problem. This was solved by removing the cell culture from the tubing after each sampling and introducing a cleaning cycle between samples. Building upon this, our study developed a process control approach for furfural substrate feeding in a fed-batch process, ensuring it does not adversely affect cell fitness. The model fed-batch process set-up consisted of two separate feeds connected to a shake flask where the cell propagation took place (Fig. 1). The main feed contained rich medium to support cell growth as well as 6.9 g/L furfural to induce the biosensor response. Additionally, a second feed containing a concentrated furfural solution (50 g/L) was connected to the cultivation through a secondary pump (Pump 2, Fig. 1). A computer program was developed to read the results from the at-line FCM analysis in real-time and communicate with an Arduino to control the action of this secondary pump.

The *TRX2*p-*yEGFP* biosensor has previously been shown to give a consistent response to furfural, indicated by a green fluorescence that correlates with furfural levels. However, this response was found to diminish over time due to yeast adaptation, occurring sometime after the addition of furfural. The transient activation of the TRX2 promoter by furfural can be attributed to several factors. Cells adapt to restore redox balance by upregulating the expression of antioxidant enzymes and increasing the production of reducing equivalents such as NADPH, with upregulation of the pentose phosphate pathway playing a crucial role. This allows the cells to neutralize the ROS generated by furfural and restore the cellular redox state. Over time, these adaptations enable the yeast cells to maintain their normal physiological functions even in the presence of furfural, leading to the observed decrease in the responsiveness of the *TRX2p-yEGFP* biosensor. At this point, yeast cells are more tolerant, and an increased amount of furfural could be added to the cultivation. This would hypothetically reinduce the *TRX2*p-*yEGFP* biosensor, which may serve as a basis for dynamic process control and maximisation of furfural feeding. Hence, the computer program was designed to start the secondary pump upon a decrease of the *TRX2*p-*yEGFP* biosensor output and study a potential reinduction of the biosensor response.

Successful implementation of the dynamic control strategy requires an automated gating method for the FCM data to obtain key input variables for the pump activation function. Accordingly, an input data processing script was written to execute the following tasks: (i) retrieve the data from the FCS files created continuously by the at-line FCM system, (ii) apply appropriate gating strategy to identify subpopulations, (iii) calculate key variables describing subpopulations, (iv) calculate the slope of the evolution of the parameters over time, and (v) use the calculated slope values to initiate or stop a peristaltic pump.

**Fig. 2.**
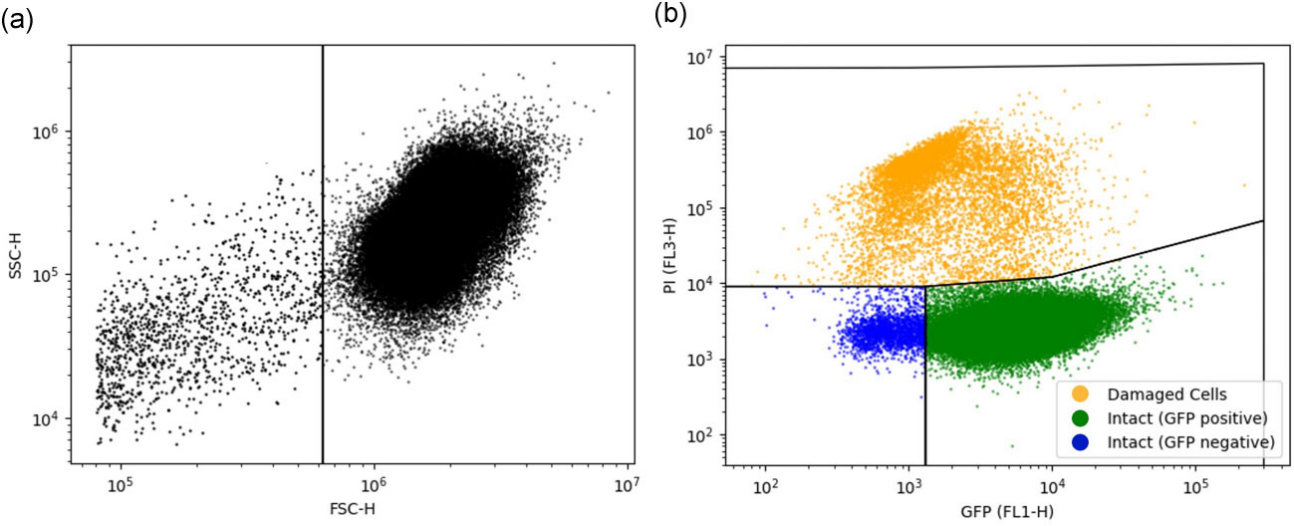
First and second step of the automated gating procedure. (a) Yeast cells (right area) are gated based on SSC-H and FSC-H. (b) A representative fcm data of a yeast population using the *TRX2*p-*yEGFP* biosensor and PI staining. Damaged cells are represented in yellow (upper area), Intact cells which are GFP positive are green (lower right area) while the intact GFP negative are blue (lower left area). FSC-H, forward scatter height; SSC-H, side scatter height; GFP, green fluorescent protein; and PI, propidium iodide.

Data were retrieved in the FCS format and converted to python files using the “FlowCytometryTools” package. Then, the yeast population was separated from background noise debris by adding a static threshold gate in the forward scatter height as indicative of particle size (Fig. 2a). Events below this threshold were considered debris and removed from further calculations. The remaining events were plotted in a scatterplot using GFP fluorescence (FL1-H) against PI fluorescence (FL3-H) (Fig. 2b). This plot allowed the distinction between PI-stained cells, referred to as “damaged” and unstained cells, referred to as “intact”. Further classification of the “intact” cells into “GFP positive” or “GFP negative” based on their GFP fluorescence was possible. The results obtained by the developed program were validated by comparison to those obtained using FlowJo (Supplementary Table 1 and Supplementary Fig. 1).

To determine if the secondary feed should be initiated, the slope of mean fluorescence intensity (MFI) GFP (FL1-H) obtained from the last three measured data points was used. An exponential moving average (EMA) was used to avoid possible outliers. EMA differs from the standard moving average by putting more weight on recent datapoints to describe the current trend. When the MFI GFP (FL1-H) slope from the three latest data points was negative, the Arduino started the secondary pump for additional furfural solution at 1 mL/hr. However, due to the inhibitory effects of furfural, there was a risk of an increase in damaged cells as indicated by PI staining. To avoid this, a function was added to the control program to stop the pump based on the number of damaged cells, which can be determined by the percentage of PI-stained cells. Thus, the percentage of PI-stained cells was used as a safeguard and the pump was stopped if the PI slope was positive or the average number of PI-stained cells was over 35%.

**Fig. 3.**
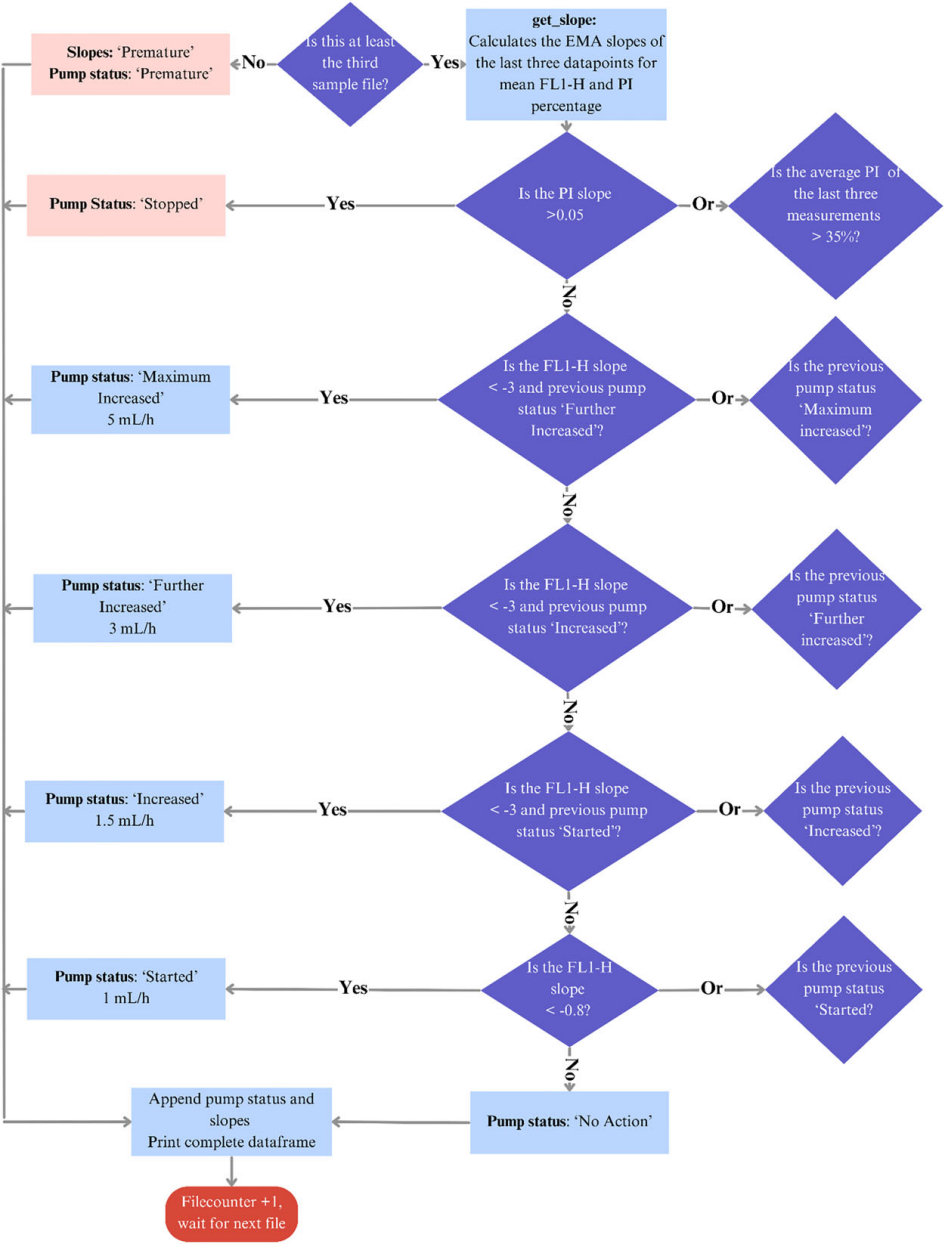
Decision-making flow chart of the program developed to initiate or stop the pump for the additional furfural solution.

After an initial batch phase, the fed-batch was initiated by starting the primary feed pump. After the initial induction, the program initiated pumping the secondary feed at an initial feeding rate of 1 mL/hr. To allow for further increase in feeding, the program was designed to implement a stepwise increase in flow rate from 1 to 1.5, 3, and 5 mL/hr of the secondary feed if a decrease in fluorescence was observed with the previous flow rate. A flow chart describing the developed computer program can be found in Fig. 3.

### Assessment of Automated Control System for Fed-Batch Cultivations

The functionality of the computer program was assessed in an automated fed-batch cultivation process with the strain TMBRP011. First, the response of the *TRX2*p-*yEGFP* biosensor was monitored in a F-FR of rich medium and 6.9 g/L furfural (F-FR), that is, without automated propagation control. In a second set of experiments, the developed computer program was initiated and the response of the *TRX2*p-*yEGFP* biosensor was used to control the addition of a concentrated furfural solution containing 50 g/L furfural by a secondary pump (SC-FR). These experiments allowed the comparison between F-FR and SC-FR cultivations.

**Fig. 4.**
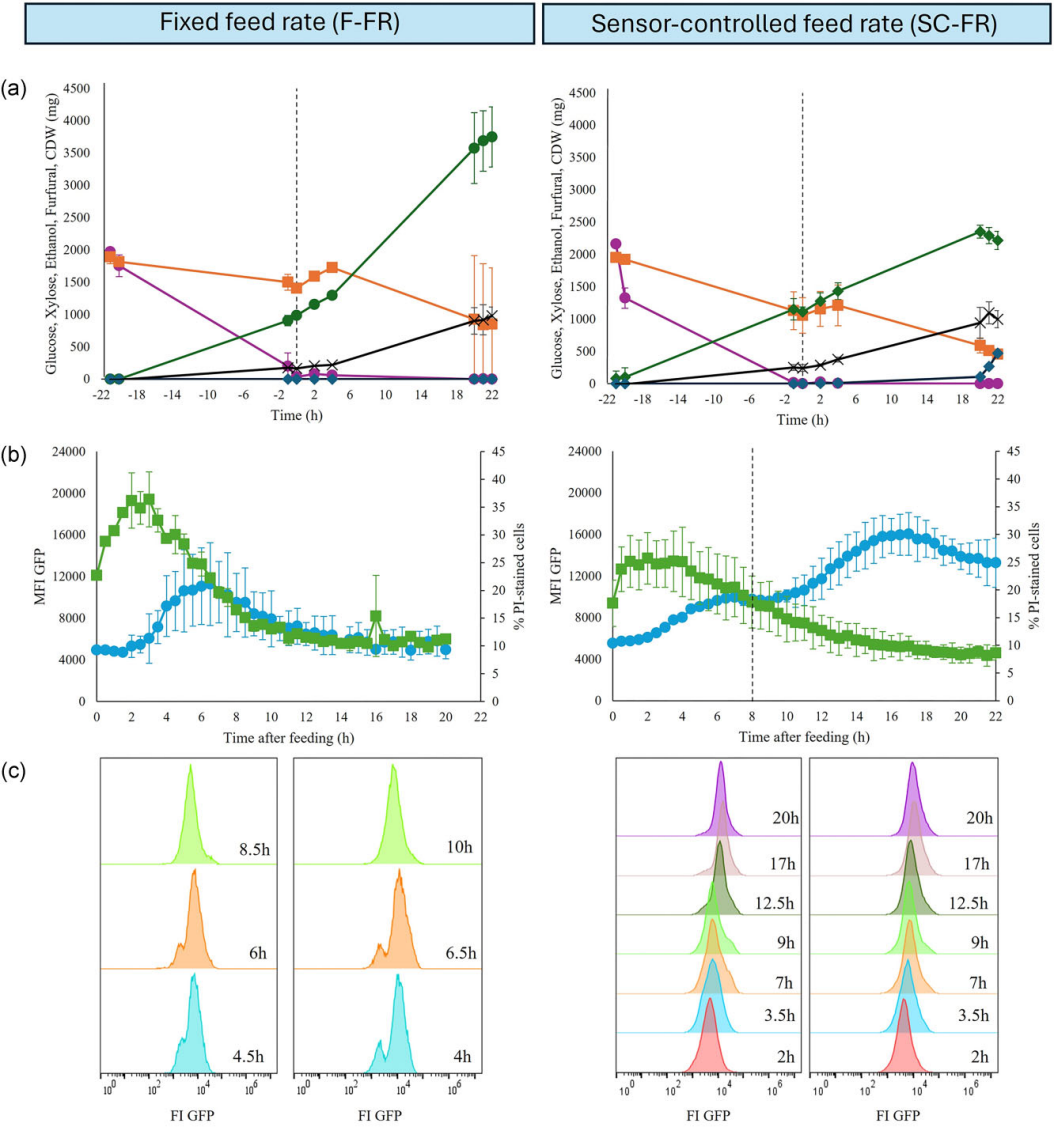
Cultivation profile of the strain TMBRP011 cultivated in fed-batch mode showing amounts of glucose (●), xylose (■), ethanol (▲), furfural (♦), and cell dry weight (×) (a). Time 0 hr corresponds to the beginning of the feeding phase which is marked with a dashed line. Mean fluorescence intensity (MFI) of GFP (●) and percentage of PI-stained cells (■) (b). Histogram showing the distribution of biosensor response in the cell population at different representative time points after feeding (c). The graphs on the left correspond to fixed feed rate fed-batch experiments (F-FR), while the ones on the right correspond to sensor-controlled feed rate fed-batch cultivations (SC-FR). Two different biological replicates for each set of experiments are shown.

Glucose was fully consumed in the initial batch phase (ca. 21–22 hr), and the feeding was subsequently initiated (Time 0, Fig. 4a). In the F-FR cultivations, an initial increase in GFP fluorescence was observed after the feeding was started (Fig. 4b, left). However, after reaching its peak, the fluorescence intensity decreased to its initial level (Fig. 4b, left) confirming the results obtained in (Perruca Foncillas et al., 2023) when furfural-containing (6 g/L) wheat straw hydrolysate was used. In the SC-FR cultivations, the same response from the biosensor was observed at the beginning of the feeding phase (Fig. 4b, right). However, after ca. 8 hr of feeding, the secondary pump controlling the additional feed of furfural was automatically initiated using the developed control program. This point corresponded with a decrease in the induction rate of the biosensor response (Fig. 4b, right). Upon initiation of the additional furfural feed, a new increase in fluorescence was observed (Fig. 4b right). This second induction peak reached its highest point around 17 hr after the feeding phase was initiated and it started decreasing again. At this point, the feeding rate of the secondary pump was increased stepwise from 1 mL/hr up to 5 mL/hr in the final stage, but no further induction of the biosensor was observed before reaching the end of the cultivation (Fig. 4b, right). This final increase in the feeding rate resulted in the accumulation of furfural in the medium (Fig. 4a, right), indicating that the addition of furfural exceeded the detoxification capabilities of the yeast cells. In total, 1985 ± 354 mg furfural was added to the fermenter with the automated control system, which is 2.3-fold higher amount than the fixed rate cultivation.

Although higher amounts of furfural were added in the SC-FR cultivations compared to the F-FR cultivations, no effects were observed in the evolution of the number of PI-stained cells over time (Fig. 4b). Both F-FR and SC-FR cultivations showed an increase in the percentage of PI-stained cells upon the initial furfural exposure reaching ca. 30%–40% at its highest point after ca. 3 hr of feeding and decreasing afterwards plateauing at ca. 10% of the population (Fig. 4b). In terms of heterogeneity in the biosensor response, a small subpopulation showing lower induction of the biosensor response was observed in the F-FR cultivations (Fig. 4c, left). This subpopulation corresponded to PI-stained cells (Supplementary Fig. 2) suggesting that membrane-damaged or even dead cells did not respond to the presence of furfural. Amongst the remaining unstained cells, a homogenous fluorescence signal was observed. Interestingly, no subpopulations were observed in the SC-FR cultivations in either of the two induction stages (Fig. 4c, right).

The additional introduction of furfural in the SC-FR fed-batches did not affect the growth of the cells, indicating that there was no negative impact on the physiological fitness of the cells. The biomass yield of SC-FR cultivations was 0.107 ± 0.014 (g CDW/g sugars), which was very similar to the 0.113 ± 0.001 (g CDW/g sugars) obtained in the F-FR cultivations. No glucose was measured in the medium (Fig. 4a), indicating that all the glucose provided by the feed was immediately consumed. In addition, xylose was nearly depleted by the end of the cultivations (Fig. 4a). A decrease in ethanol production was observed in the SC-FR cultivations (Fig. 4a).

### Impact of the Yeast Propagation Method on Bioethanol Production

Short-term adaptation to inhibitors has previously been shown to improve lignocellulosic fermentation (Nielsen et al., 2015; Narayanan et al., 2017; Almeida et al., 2023; Perruca Foncillas et al., 2023). To know whether more challenging furfural adaptation conditions had any positive impact on the ethanol yield and/or productivity, the performance of the yeast cells generated with the help of the developed automated control system was evaluated in an anaerobic bioethanol production process. Cells were propagated again in F-FR cultivations and SC-FR cultivations and used for inoculation of anaerobic fermentation vials containing wheat straw hydrolysate with inhibitor levels corresponding to those in a 10% WIS SSF.

Based on the results shown above, cells were collected at their highest induction point corresponding to 17 hr after the feeding was started (Fig. 4b, right). At this point, SC-FR-propagated cells had an MFI of 16 300 compared to the 10 300 MFI for F-FR-propagated cells. Similar results to those shown above for F-FR and SC-FR cultivations were obtained during the propagation (Supplementary Fig. 3).

**Fig. 5.**
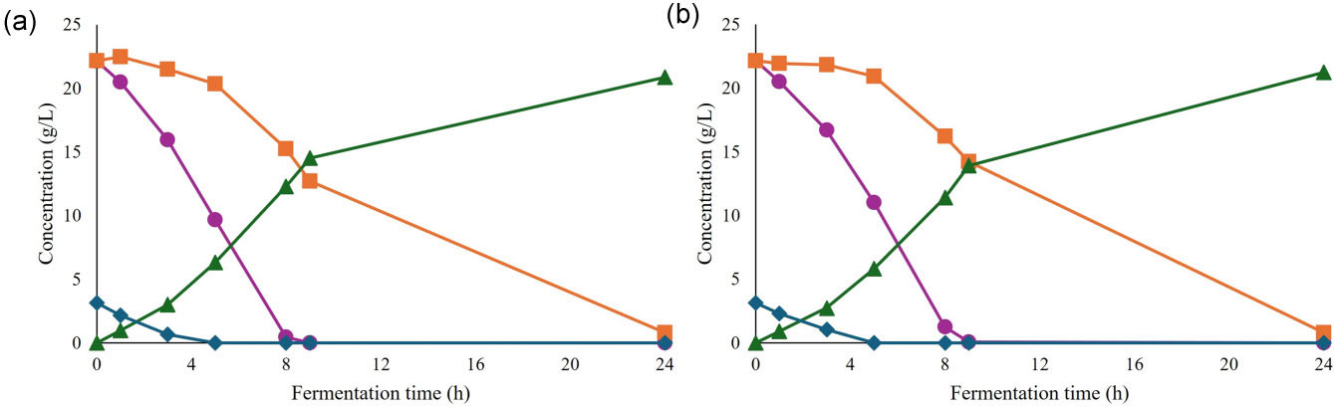
Fermentation profile of the strain TMBRP011 in 10% WIS equivalent of wheat straw hydrolysate for cells propagated in F-FR and SC-FR fed-batch cultivations. The concentrations of glucose (●), xylose (■), ethanol (▲), and furfural (♦) for F-FR-propagated cells (a) and SC-FR-propagated cells (b) are shown.

During the following fermentation on wheat straw hydrolysate, similar results were obtained for F-FR and SC-FR-propagated cells, demonstrating the equal performance of the two yeast cultures (Fig. 5). Glucose was depleted after 9 hr of fermentation, followed by xylose depletion after 24 hr (Fig. 5). Both F-FR and SC-FR exhibited similar ethanol production, reaching their maximum concentration (approximately 20 g/L) within just 24 hr of fermentation. At this point, the ethanol yield was 0.48 (g ethanol/g sugars) for F-FR and 0.49 (g ethanol/g sugars) for SC-FR, which correspond to 94.2% and 95.8% of the theoretical maximum ethanol yield, respectively. No changes in metabolite concentrations were observed until the completion of the 96-hr fermentation (data not shown). These results suggest that the different propagation modes had no effect on the fermentation performance of the cells.

## Discussion

The use of real-time monitoring and control strategies in microbial cultivations is crucial to obtaining efficient bioprocesses. In this study, an automated control system based on the response of a transcription factor-based biosensor using FCM was used to adjust the feed of furfural in a fed-batch cultivation process.

The developed system was successfully implemented in fed-batch cultivations of the industrial *S. cerevisiae* strain TMBRP011, engineered for xylose utilization and carrying the *TRX2*p-*yEGFP* biosensor for redox imbalance (Perruca Foncillas et al., 2023). A decrease in MFI from the *TRX2*p-*yEGFP* biosensor was associated with an adaptation from the cells to the furfural levels as they were no longer suffering from NADPH deficiency. Thus, when a negative slope in MFI over time was observed, the amount of furfural could be increased through the addition of a second feed to re-induce the cell response. The frequency of PI-stained cells was also considered as a parameter for the decision-making of the program since too high concentrations of furfural are cytotoxic. However, this so-called “safeguard function” was not activated under the conditions applied for the cultivations described herein. With the combination of these two parameters, the control system was able to successfully generate a second induction of the biosensor response in the culture.

The activation of the secondary pump resulted in up to 2.3 times higher amounts of furfural being introduced into the SC-FR cultures compared to the F-FR cultivations. The increased amounts of furfural did not result in an increase in PI-stained cells or furfural accumulation in the medium. This suggests that the cells have an increased tolerance towards furfural and can detoxify higher quantities by NADPH-dependent reduction to furfuryl alcohol (Almeida et al., 2008). The decrease in fluorescence observed after 17 hr of feeding indicates that the cells were adapted when the furfural feed was added at 1 mL/hr. The lack of reinduction after increasing the feed rate up to 5 mL/hr could indicate an optimal state in the cell culture where no NADPH deficiency is triggered by the presence of furfural anymore. For example, the cells could have reached their maximum capacity for detoxification by maximising the production of the responsible enzymes.

Since our previous study had shown a correlation between biosensor induction during propagation and better fermentation performance (Perruca Foncillas et al., 2023), we hypothesized that SC-FR-propagated cells could show better fermentation performance due to their increased induction levels at the time of cell collection. However, no differences between F-FR and SC-FR in fermentation performance were observed, indicating similar cell fitness for both cultures regardless of the amount of supplemented furfural.

One of the most powerful features of the developed control system is the fact that its response is based on the redox state of the cells, an intracellular property, as opposed to the use of external parameters such as pH or off-gas composition (Taherzadeh et al., 2000; Nilsson et al., 2001) or the concentration of external metabolites which is often utilized to control cultivation processes (Petersson & Lidén, 2007). Furthermore, since it is based on FCM analysis, it can be useful in processes where subpopulations are expected.

Although this system was developed to meet the specific needs of this project, it could be modified to be applicable to other fed-batch cultivations. In the present study, the FL1-H and FL3-H values were used for the consequent calculations of the MFI and percentage of PI-stained cells; but, since it is based on the information gathered in the FCS file from the FCM analysis, other parameters found in those files could be used as input variables for the control system. For example, one could envision a more complex system based on the simultaneous measurement of several biosensors, each of them monitored via different fluorescent proteins (Perruca-Foncillas et al., 2022). This may be important for the control of microbial population heterogeneity in large-scale fermentation by strain and process engineering (Mu & Zhang, 2023). The system could also be applied in cases where the introduction of biosensors is not possible, that is, due to the lack of relevant biosensors or genetically modified organism (GMO) regulations. In such cases, parameters such as fluorescence from dyes or even the number of events could be used as input variables for the control system.

In the realm of bioprocessing, the convergence of synthetic biology and computational science has given rise to cybergenetics (Jones et al., 2024), a paradigm where advanced monitoring and control strategies are orchestrated through seamless integration of computational algorithms and biological systems. Our developed control system exemplifies the principles of cybergenetics by integrating real-time monitoring of gene activation using FCM with dynamic adjustment of cultivation parameters. This seamless interaction between biological sensors, computational algorithms, and control mechanisms not only optimizes bioprocess performance but also opens avenues for more sophisticated control strategies in the future.

## Supplementary Material

kuae039_Supplemental_File

## Data Availability

The data underlying this article will be shared on reasonable request to the corresponding author.
