# Supplementary material for: Automated yeast cultivation control using a biosensor and flow cytometry
Source: J Ind Microbiol Biotechnol. 2024 Oct 18;51:kuae039. doi: 10.1093/jimb/kuae039 (PMC11561399; doi:10.1093/jimb/kuae039)
Supplement: kuae039_Supplemental_File [file kuae039_supplemental_file.docx]

**Automated yeast propagation control using a biosensor and flow cytometry**

Raquel Perruca Foncillas^1^, Sara Magnusson^1^, Basel Al-Rudainy^2^, Ola Wallberg^2^, Marie F. Gorwa-Grauslund^1^, Magnus Carlquist^1^

**Affiliations**

^1^ Applied Microbiology, Department of Chemistry, Lund University, P.O. Box 124, SE-221 00 Lund, Sweden

^2^ Division of Chemical Engineering, Department of Process and Life Science Engineering, Lund University, P. O. Box 124, SE-221 00 Lund, Sweden

* Correspondence: [Magnus.carlquist@tmb.lth.se](mailto:Magnus.carlquist@tmb.lth.se)

**Keywords**

Automated real-time flow cytometry; Fermentation; Control Strategy; Heterogeneity; Synthetic biology;

Supplementary Figures

Figure S1

Figure S2

Figure S3

Supplementary Table

Table S1


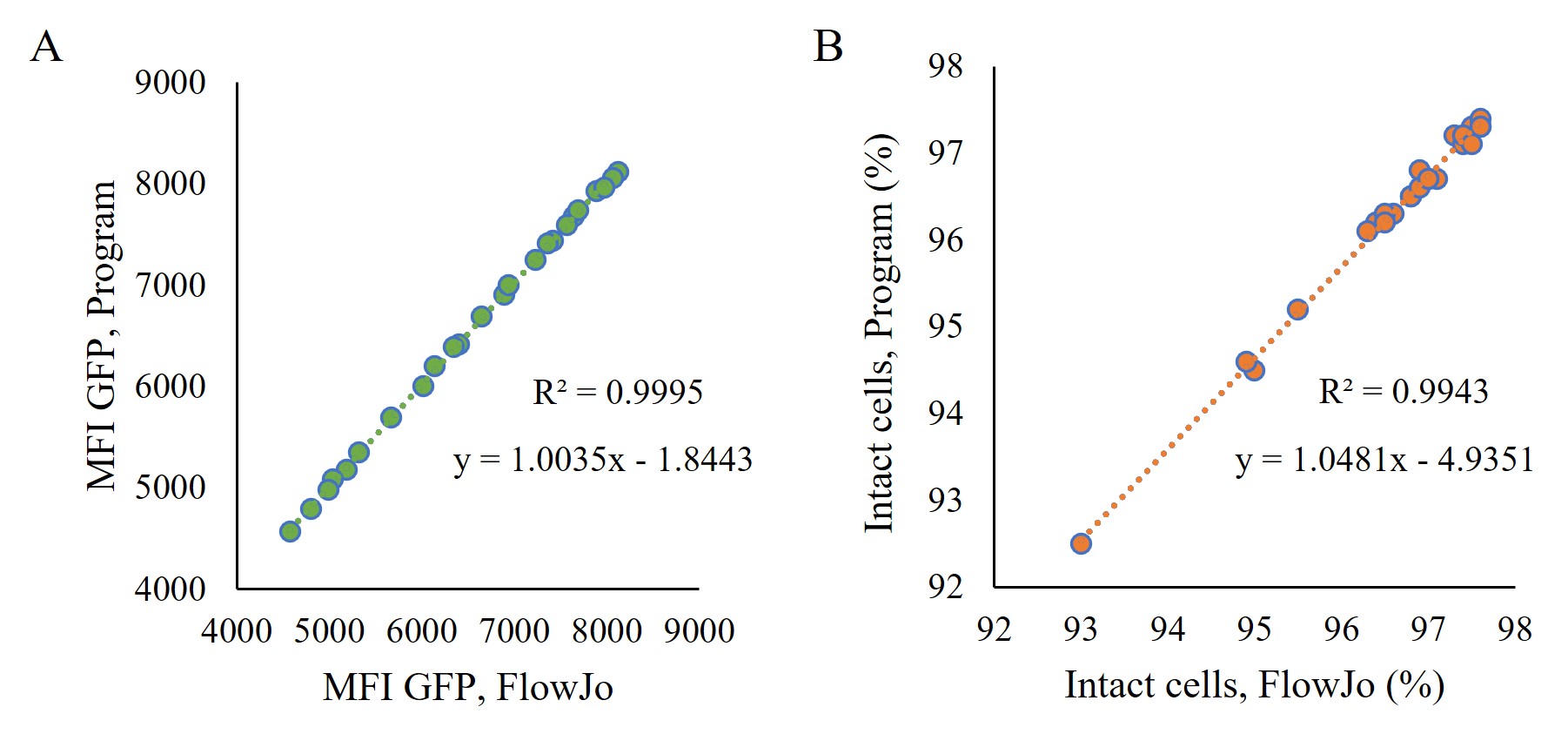


Figure S1. Comparison between the results obtained with the program automatic output and FlowJo manual output for MFI GFP (A) and percentage of unstained cells (B). A linear fitting was performed for the correlation between the methods.


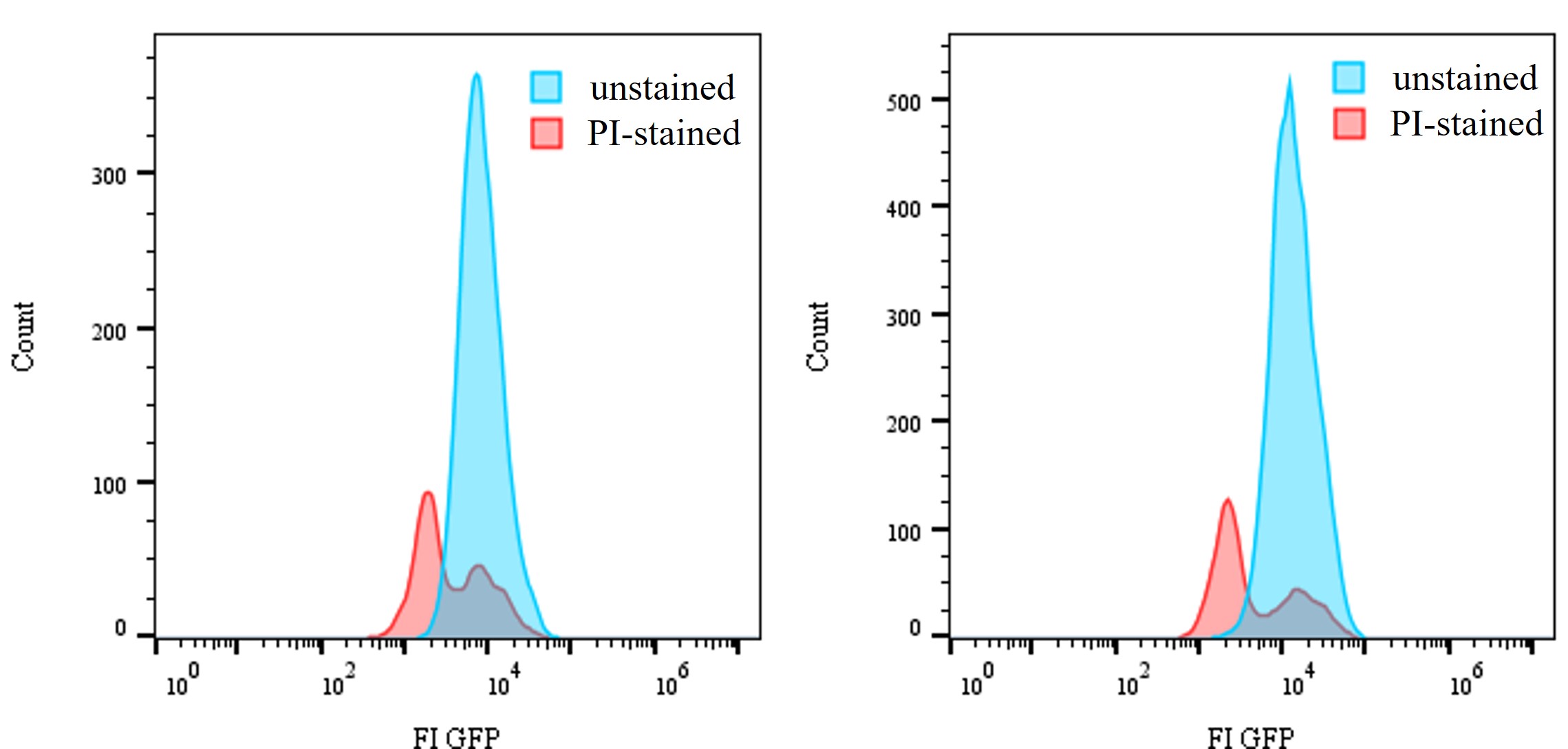


Figure S2. Histogram of GFP fluorescence of the two replicates of F-FR cultivations after 6 hours of feeding.


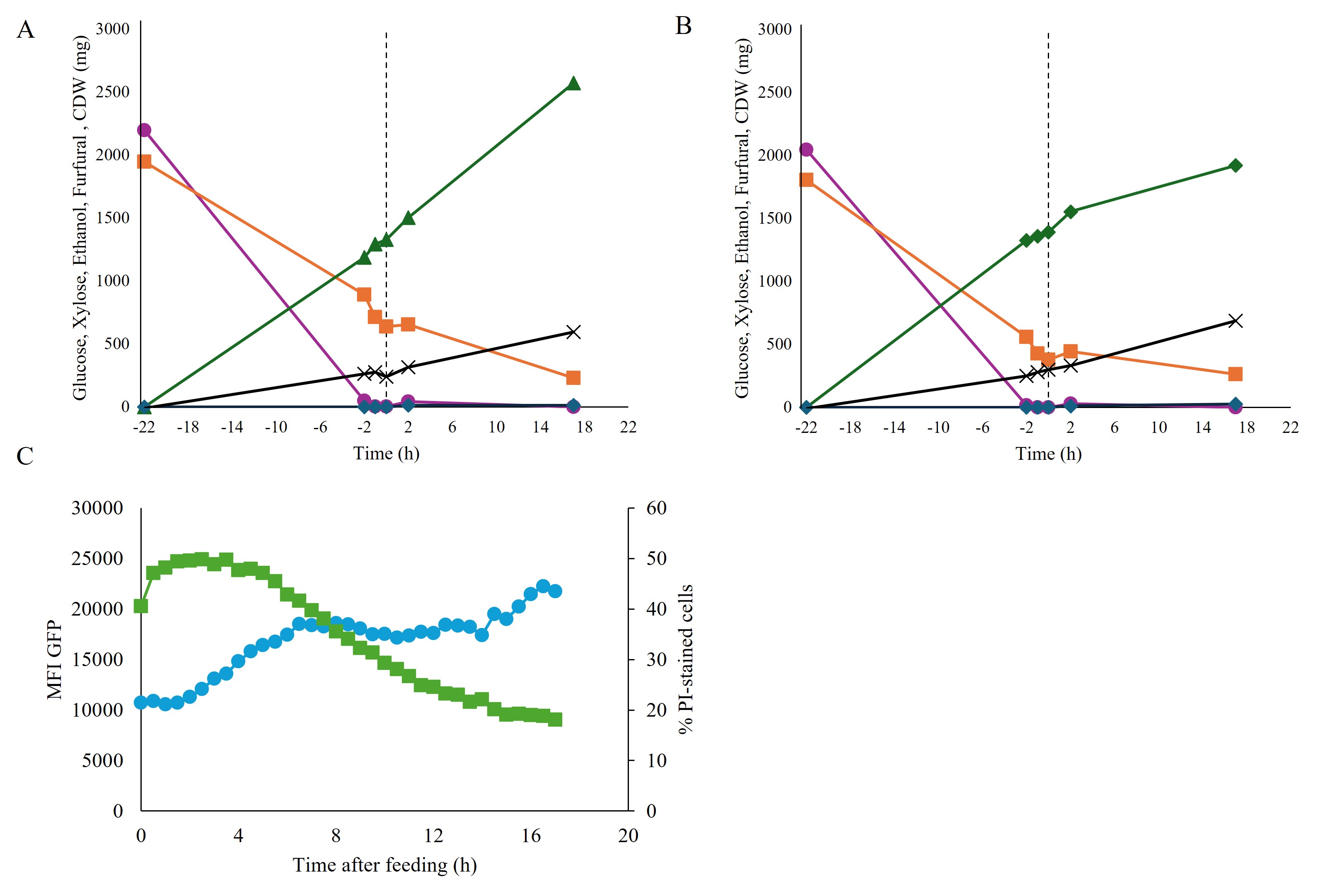


Figure S3. Cultivation profile of the strain TMBRP011 showing amounts of glucose (●), xylose (■), ethanol (▲), furfural (♦) and cell dry weight (CDW) (×) during propagation in SC-FR (A) and F-FR (B). Time 0 hours corresponds to the beginning of the feeding phase which is marked with a dashed line. Mean fluorescence intensity (MFI) of GFP (●) and percentage of PI-stained cells (■) in SC-FR (C).

Supplementary Table

Table S1. Example of the comparison between the results obtained from the gating strategy by the developed program and the results obtained with FlowJo for the same FCS file. PI: propidium iodide; MFI: mean fluorescence intensity; GFP: green fluorescence protein.

| Parameter | Developed program | FlowJo |
| --- | --- | --- |
| Number of events | 97753 | 97753 |
| Number of cells | 96511 | 95409 |
| Percentage of PI-stained cells | 92.6% | 93.0% |
| MFI GFP (Intact Cells) | 6412 | 6402 |
